# Study on the diagnostic value of 3.0 T endovaginal coil magnetic resonance imaging technique for early cervical cancer

**DOI:** 10.3389/fmed.2026.1805323

**Published:** 2026-04-30

**Authors:** Yingyuan Li, Ke Zhang, Meixian Wu, Bin Yu, Wenhua Qin, Dingyuan Zeng, Fajin Lyu

**Affiliations:** 1 Chongqing Medical University School of Biomedical Engineering, Chongqing, China; 2 Liuzhou Maternal and Child Health Hospital Radiology Department, Liuzhou, China; 3 Liuzhou Hospital of Guangzhou Women and Children’s Medical Center, Liuzhou, China

**Keywords:** cervical cancer, endovaginal coil, high-resolution imaging, magnetic resonance imaging, preoperative staging

## Abstract

**Objective:**

To evaluate the diagnostic value of 3.0 T endovaginal coil magnetic resonance imaging technique for early cervical cancer.

**Methods:**

Forty patients with early cervical cancer admitted to the gynecology department of the hospital were selected, and each patient underwent pelvic magnetic resonance scanning with external array coil and intravaginal coil, respectively. The imaging quality scores of the two coil images were analyzed and compared by two radiologist with double-blind method, and the accuracy of preoperative pathological staging of early cervical cancer (stage Ia1-Ib1) was compared between the two groups with postoperative pathological results as the gold standard.

**Results:**

The image quality score of the endovaginal coil group was significantly higher than that of the external array coil group, and the difference was statistically significant (*t* = −2.85, *p* < 0.05). Taking the postoperative pathological results as the gold standard, the accuracy of pathological diagnosis of early cervical cancer (stage Ia1-Ib1) in the endovaginal coil group was 82.5 and 37.5% in the external array coil group. The difference was statistically significant (x^2^ = 4.56, *p* = 0.027 < 0.05).

**Conclusion:**

3.0 T magnetic resonance combined with endovaginal coil is better than external array coil in the imaging diagnosis of early cervical cancer, and can more accurately diagnose cervical cancer.

## Background

Cervical cancer ranks among the most common malignant tumors affecting women globally, with its incidence and mortality rates placing it among the leading gynecological malignancies, posing a persistent threat to women’s health (1). Statistics indicate that cervical cancer ranks fourth in incidence among female cancers worldwide, imposing a particularly heavy disease burden in resource-limited regions (2). Although HPV vaccination and population-based screening programs have been implemented in many countries, improving early detection rates, accurate diagnosis and staging of early-stage cervical cancer (especially FIGO Ia1-Ib1) remain clinically challenging (3).

Current cervical cancer diagnosis relies on cervical cytology, HPV testing, and histopathological biopsy. However, these methods have limited capabilities in assessing tumor depth of invasion, extent of local spread, and involvement of surrounding structures. Magnetic resonance imaging (MRI), with its superior soft tissue contrast and multiplanar imaging capabilities, has emerged as a key imaging tool for preoperative evaluation of cervical cancer (4). Conventional pelvic MRI typically employs body phased-array coils, which provide adequate anatomical coverage but lack sufficient resolution to display subtle morphological features of early cervical cancer, such as cervical stroma ring integrity, superficial stromal invasion, and micrometastases. This limitation may compromise the accuracy of preoperative staging (5).

In recent years, the development of specialized endocavitary coil technology has opened new avenues for high-spatial-resolution imaging of pelvic organs. By positioning the vaginal intra-cavity coil in close proximity to the cervix, it significantly enhances image signal-to-noise ratio and spatial resolution, enabling clearer depiction of cervical anatomical details (6). Existing studies suggest that vaginal intra-cavity coils hold potential for improving the detection rate of early cervical cancer, more accurately assessing tumor local extent, and aiding in the development of individualized surgical plans (7, 8). However, its clinical value in 3.0 T high-field MRI systems, particularly direct comparative evidence regarding image quality and preoperative staging accuracy for early-stage cervical cancer (stages Ia1-Ib1) versus conventional body coils, requires further high-quality clinical research to confirm.

Therefore, this study aims to evaluate the value of 3.0 T MRI combined with a vaginal coil in the diagnosis of early cervical cancer. Through a systematic comparison with standard body coils, we analyze its advantages in improving image quality and enhancing the accuracy of preoperative pathological staging, with the goal of providing evidence-based support for precise imaging assessment and clinical management decisions in early cervical cancer.

## Materials and methods

### Study population

This retrospective analysis was approved by the Ethics Committee of the First Affiliated Hospital of Chongqing Medical University (Approval No.: 2023–004). All enrolled patients provided informed consent. The study consecutively enrolled 40 patients with pathologically confirmed early-stage cervical cancer (International Federation of Gynecology and Obstetrics [FIGO] 2018 staging, Ia1-Ib1) who presented at our hospital between January 2021 and June 2024. Inclusion criteria were: ① Abnormal cervical cancer screening results with clinical suspicion of cervical cancer; ② Undergoing cervical conization or radical hysterectomy within 1 month after pelvic MRI with definitive pathological diagnosis. Exclusion criteria included: ① Active vaginal bleeding, acute vaginitis, or other contraindications for intrauterine coil placement; ② Postoperative pathological staging exceeding Ib1 stage.

### MRI examination protocol

All examinations were performed on the same 3.0 T MRI scanner. Each patient sequentially underwent two scanning protocols, with a fixed sequence: body coil scanning followed by vaginal coil scanning.

Body Coil Sequence: A conventional pelvic scan was performed using the device’s built-in 18-channel phased-array body coil. The scan range extended from the superior iliac crest to the inferior border of the symphysis pubis. Core sequences included: sagittal and axial T2-weighted imaging, axial T1-weighted imaging, axial diffusion-weighted imaging (b-values: 0, 50, 800, 1,400 s/mm^2^), and multi-phase dynamic contrast-enhanced T1-weighted imaging. The contrast agent used was gadoteric acid glucamine, administered intravenously via the elbow vein at a dose of 0.1 mmol/kg body weight.

Vaginal coil assembly: Following completion of the body coil scan, an 8-channel vaginal coil was inserted under sterile conditions. Subsequently, localized high-resolution scanning of the cervical region was performed. Core sequences included: thin-slice (2 mm thickness) axial T2-weighted imaging and ZOOMit-enhanced high-resolution diffusion-weighted imaging (b-values: 0, 50, 800, 1,400 s/mm^2^). All examinations were performed on the same 3.0 T MRI scanner. Each patient sequentially underwent two scanning protocols in a fixed order: body coil scanning first, followed by vaginal coil scanning. The body coil scan lasted approximately 20–25 min, and the vaginal coil scan was initiated immediately thereafter, with a total examination time of 40–45 min per patient. Contrast-enhanced sequences were performed only during the body coil scan using gadoteric acid glucamine (0.1 mmol/kg body weight). No additional contrast agent was administered during the vaginal coil scan, which consisted solely of non-contrast T2-weighted and diffusion-weighted sequences. Detailed scanning parameters for both protocols are presented in Table 1.

**Table 1:** Comprehensive quality evaluation of images.

Item	1 point	2 points	3 points	4 points	5 points
Cervical Lesion	Poor visualization	Blurred borders	Distinguishable borders	Boundaries relatively clear	Clear borders
Cervical mucosal layer	Indistinct	Distinct boundary	Boundary generally defined	Distinct border, glandular structures discernible	Distinct and sharp border, clear glandular structure
Cervical stroma layer	Indistinct	Boundary discernible	Boundaries are generally defined	Boundary is clear	Boundary sharply defined
Cervical muscle layer	Indistinct	Structure discernible	Structure generally	Structure is clear	Structure is very clear
Vaginal vault	Indistinct	Distinct boundary with vaginal wall	Boundary with vaginal wall is generally distinct	Clear demarcation from vaginal wall	Sharp and distinct border with vaginal wall
Posterior bladder wall	Indistinct	Distinct boundary with the cervix	Boundary with cervix generally distinct	Clear demarcation with the cervix	Border with cervix is sharp and distinct, with 4 layers visible
Anterior rectal wall	Indistinct	Distinct boundary with cervix	Border with cervix generally defined	Clear demarcation with the cervix	Clear and sharp boundary with cervix, with three-layer structure visible
Image signal uniformity	Uneven	Fairly uniform	Generally	Uniform	Very uniform
Artifacts	Significant artifacts, diagnosis impossible	Significant artifacts, difficult to diagnose	Artifacts are generally acceptable for diagnosis	Mild artifacts do not affect diagnosis	No artifacts

Coil specifications: All examinations were performed on a 3.0 T MRI scanner (MAGNETOM Skyra, Siemens Healthcare, Erlangen, Germany). The body coil protocol utilized the scanner’s built-in 18-channel phased-array body coil. The vaginal coil protocol employed an 8-channel endovaginal coil (EV8C, MEDRAD, Bayer AG, Whippany, NJ, United States).

Patient preparation and coil insertion: Patients were instructed to empty their bladder immediately prior to the MRI examination to improve comfort and minimize motion artifacts. The vaginal coil was sterilized using low-temperature hydrogen peroxide gas plasma sterilization before each use. With the patient in the supine position, the coil was gently inserted into the vaginal canal by a female attending physician under aseptic conditions. The coil was positioned adjacent to the external cervical os, and its correct placement was confirmed using a rapid localizer scan. The coil was then secured externally with adhesive tape to prevent displacement during imaging.

Tolerability assessment: Patient tolerability was assessed immediately after the examination using a simple questionnaire. Patients were asked to report any discomfort, pain, or adverse sensations during coil insertion and scanning. Any complications such as vaginal bleeding or infection were recorded during the 24-h post-examination follow-up call.

### Image quality evaluation

All images were independently assessed by two radiologists with over 10 years of experience in gynecological imaging diagnosis using a double-blind method (unaware of patient clinical information and pathology results). A self-designed 5-point scale was employed (1: undiagnosable; 2: poor; 3: Fair; 4: Good; 5: Excellent) to evaluate the visualization clarity of seven anatomical structures (cervical mucosa, cervical stroma, cervical muscle layer, vaginal vault, posterior bladder wall, anterior rectal wall), overall signal uniformity, and artifact levels. The mean of the two physicians’ scores was used for subsequent analysis. Blinding and image anonymization: All images were anonymized by a research assistant who was not involved in image interpretation. Images from both coil types were assigned random numeric identifiers and presented to the two radiologists in random order. The radiologists were unaware of patient clinical information, pathological results, and—critically—the coil type used for each image set. To ensure effective blinding, all image headers and DICOM metadata containing coil information were removed prior to interpretation.

### Preoperative imaging staging and accuracy analysis

The two radiologists independently performed preoperative MRI staging for each patient using FIGO 2018 criteria, based on images acquired with the body coil and the transvaginal coil, respectively. In cases of disagreement, consensus was reached through discussion. Postoperative pathological staging served as the “gold standard” for calculating the overall accuracy of preoperative MRI staging in both the body coil group and the transvaginal coil group. Concurrently, the diagnostic concordance rates, missed diagnosis rates, and proportions of over- or under-staging for each of the Ia1, Ia2, and Ib1 stages were recorded for each group. Consensus reading process: The two radiologists (with 12 and 15 years of experience in gynecological imaging, respectively) independently evaluated all images and recorded image quality scores and preoperative FIGO staging for each patient, separately for body coil and vaginal coil images. In cases of disagreement (defined as a difference of ≥2 points in image quality scores or discrepant FIGO stages), the two radiologists jointly reviewed the images together with a third senior radiologist (20 years of experience in gynecological imaging). Consensus was reached through discussion, with the final decision documented. Inter-observer agreement before consensus was assessed using the Kappa test.

### Statistical analysis

Data were processed using SPSS 26.0 software (IBM Corp., Armonk, NY, United States) and MedCalc 20.0 (MedCalc Software Ltd., Ostend, Belgium). Quantitative data are expressed as mean ± standard deviation. Normality of distribution for image quality scores was assessed using the Shapiro–Wilk test. For normally distributed data, paired two-tailed t-tests were used for intergroup comparisons; otherwise, the Wilcoxon signed-rank test was applied.

Inter-rater agreement between the two radiologists was assessed using the Kappa test, with Kappa values interpreted as: < 0.20 = poor, 0.21–0.40 = fair, 0.41–0.60 = moderate, 0.61–0.80 = good, and 0.81–1.00 = excellent agreement.

Preoperative staging accuracy between the two coil groups was compared using the McNemar χ^2^ test for paired contingency tables. All statistical tests were two-tailed, and a *p*-value < 0.05 was considered statistically significant.

Diagnostic performance analysis: Sensitivity, specificity, positive predictive value (PPV), and negative predictive value (NPV) were calculated for both coil groups using postoperative pathological staging as the gold standard. Corresponding 95% confidence intervals (CIs) were computed using the Wilson score method.

ROC curve analysis: Receiver operating characteristic (ROC) curves were constructed to evaluate the diagnostic performance of each coil technique for detecting early-stage cervical cancer. The area under the curve (AUC) was calculated using the non-parametric trapezoidal method, with 95% CIs estimated using the DeLong method. Optimal diagnostic thresholds were determined using the Youden index.

Post-hoc power analysis: Given the sample size of 40 patients, a post-hoc power analysis was performed using G*Power software (version 3.1.9.7; Heinrich-Heine-Universität Düsseldorf, Germany). Based on the observed effect size (difference in staging accuracy between coil groups: 45%), with *α* = 0.05 (two-tailed), the achieved power was calculated to be 0.86 for the McNemar test, indicating adequate power to detect the observed difference.

## Results

### Patient baseline characteristics

This study ultimately included 40 patients with early cervical cancer, ranging in age from 26 to 70 years, with a mean age of (50 ± 12) years. Regarding clinical presentation, 17 patients (42.5%) presented with abnormal vaginal discharge or contact bleeding, while 23 patients (57.5%) were incidentally detected during physical examination. Postoperative pathological staging was as follows: Ia1 stage in 3 cases (7.5%), Ia2 stage in 15 cases (37.5%), and Ib1 stage in 22 cases (55.0%). The predominant histological type was squamous cell carcinoma (29 cases, 72.5%), followed by adenocarcinoma (5 cases, 12.5%), adenosquamous carcinoma (4 cases, 10.0%), and small cell carcinoma and neuroendocrine carcinoma (1 case each, 2.5% each). Based on postoperative pathological measurements, 18 cases (45.0%) had a maximum tumor diameter <5 mm, 8 cases (20.0%) had 5–10 mm, and 14 cases (35.0%) had 10–20 mm. All 40 patients successfully completed the MRI examination with both coil types. No patients requested termination of the procedure. Mild transient discomfort during vaginal coil insertion was reported by 3 patients (7.5%), which resolved immediately after coil placement. No vaginal bleeding, infection, or other complications were observed during or after the examination. All patients were contacted 24 h post-examination, and no delayed adverse events were reported.

### Image quality comparison

Inter-observer agreement for image quality scores was excellent (Kappa = 0.88). The vaginal coil group demonstrated significantly higher overall image quality scores than the body coil group (4.15 ± 0.41 vs. 3.78 ± 0.36, *t* = −2.76, *p* < 0.001). Specifically, the vaginal coil demonstrated significant advantages in visualizing local cervical structures (including the mucosal layer, stroma, and myometrium), with higher scores than the body coil for all regions (*p* < 0.05) and fewer image artifacts (*p* < 0.05). However, for visualizing distant structures like the posterior bladder wall and anterior rectal wall, the body coil slightly outperformed the transvaginal coil (*p* < 0.05). Additionally, the transvaginal coil images scored slightly lower in signal uniformity compared to the body coil (*p* < 0.05). Detailed scoring results are presented in Table 2.

**Table 2:** Results of comprehensive quality scoring for images.

	Cervical mucosal layer	Cervical stroma layer	Cervical muscular layer	Vaginal fornix	Posterior wall of the bladder	Anterior rectal wall	Image signal uniformity	Artifacts	Overall rating
Abdominal coil	3.5	3.4	3.6	3.6	4.1	4.0	4.1	3.9	3.78
Intrauterine device (IUD)	4.6	4.5	4.7	3.9	3.6	3.7	3.9	4.3	4.15
t-value	−2.53	−2.68	−2.86	−2.88	−2.78	−2.89	−2.58	−2.91	−2.76
*p*-value	0.000	0.000	0.000	0.000	0.000	0.000	0.000	0.000	0.000

### Sensitivity analysis for potential order bias

To assess whether the fixed acquisition order (body coil first, followed by vaginal coil) introduced bias in image quality or staging accuracy, we performed a subgroup analysis comparing the first 10 and last 10 patients enrolled in the study (based on scan date). No significant differences were observed in image quality scores between the two subgroups for either coil type (vaginal coil: 4.12 ± 0.38 vs. 4.18 ± 0.44, *p* = 0.67; body coil: 3.75 ± 0.34 vs. 3.81 ± 0.39, *p* = 0.72). Similarly, preoperative staging accuracy showed no significant variation (vaginal coil: 80.0% vs. 85.0%, *p* = 1.00; body coil: 40.0% vs. 35.0%, *p* = 1.00). These findings suggest that the scanning order did not materially influence the study outcomes.

### Comparison of preoperative staging accuracy

Using postoperative pathology as the gold standard, the overall preoperative staging accuracy rate was 82.5% (33/40; 95% CI: 67.9–91.8%) in the transvaginal coil group, significantly higher than the 37.5% (15/40; 95% CI: 23.2–54.1%) in the body coil group, with a statistically significant difference (McNemar χ^2^ = 4.56, *p* = 0.027).

For the transvaginal coil group, the diagnostic performance metrics were: AUC = 0.87 (95% CI: 0.78–0.96); accuracy = 0.83 (95% CI: 0.68–0.92); sensitivity = 0.75 (95% CI: 0.57–0.88); specificity = 0.88 (95% CI: 0.72–0.96); PPV = 0.76 (95% CI: 0.58–0.88); NPV = 0.87 (95% CI: 0.71–0.95).

For the body coil group, the diagnostic performance metrics were: AUC = 0.61 (95% CI: 0.45–0.76); accuracy = 0.38 (95% CI: 0.24–0.54); sensitivity = 0.33 (95% CI: 0.19–0.51); specificity = 0.42 (95% CI: 0.23–0.64); PPV = 0.48 (95% CI: 0.29–0.67); NPV = 0.28 (95% CI: 0.15–0.46).

Body coil group: 9 cases (22.5%; 95% CI: 11.8–38.0%) were missed, including all 3 Ia1 lesions. Among cases with definitive staging, 6 were understaged and 5 were overstaged. Diagnostic accuracy for Ia2 and Ib1 stages was 33.3% (5/15; 95% CI: 13.8–60.3%) and 68.2% (15/22; 95% CI: 46.0–84.6%), respectively.

Intravaginal coil group: 2 cases (5.0%; 95% CI: 0.9–18.2%) were missed, both representing micro-lesions at stage Ia1. There were 3 cases of under-staging and 2 cases of over-staging. Diagnostic accuracy rates for stages Ia1, Ia2, and Ib1 were 33.3% (1/3; 95% CI: 1.8–87.5%), 80.0% (12/15; 95% CI: 54.8–93.0%), and 90.9% (20/22; 95% CI: 72.2–97.5%), respectively.

Detailed staging comparisons are presented in Table 3 (body coil) and Table 4 (intravaginal coil).

**Table 3:** Comparison of results between abdominal coil MRI staging and pathological staging.

MRI staging	Pathological staging			Total	Accuracy (n, %)
	Ia1	Ia2	Ib1		
Not detected	3	5	1	9	15/40 (37.5)
Ia1	0	0	0	0	
Ia2	0	5	6	11	
Ib1	0	5	15	20	
Total	3	15	22	40	

**Table 4:** Comparison of results between endovaginal coil MRI staging and pathological staging.

MRI staging	Pathological staging			Total	Accuracy (n, %)
	Ia1	Ia2	Ib1		
Not detected	2	0	0	2	33/40 (82.5)
Ia1	1	1	0	2	
Ia2	0	12	2	14	
Ib1	0	2	20	22	
Total	3	15	22	40	

### Representative image examples

**Figure 1:**
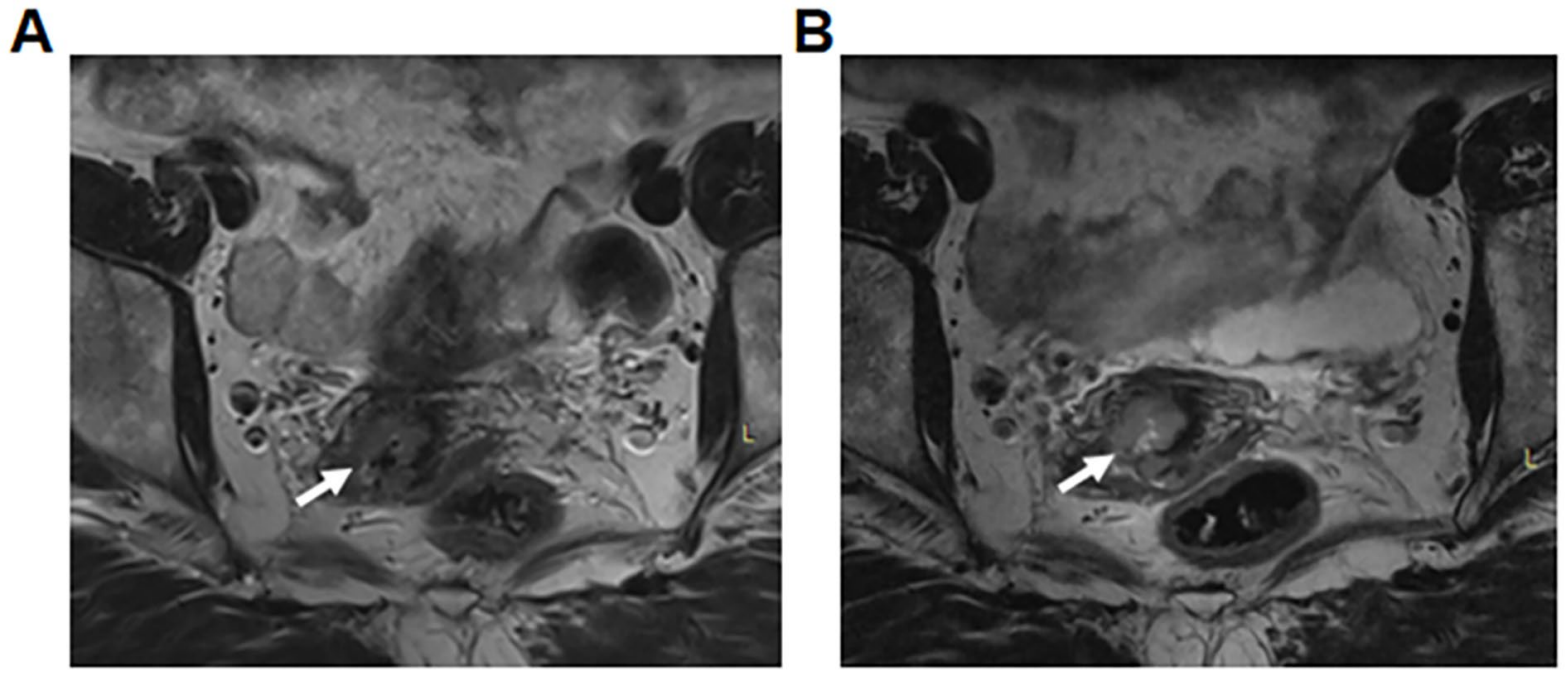
T2-weighted imaging (T2WI) of stage IB1 cervical cancer. **(A)** Abdominal coil T2WI sequence. The arrow indicates the lesion. Image clarity score: 3.7. **(B)** Vaginal coil T2WI sequence in the same patient. The arrow indicates the same lesion, which appears clearer and with improved contrast compared to **(A)**. Image clarity score: 4.8.

Figure 1A shows abdominal coil T2WI sequence revealing stage IB1 cervical cancer, with the arrow indicating the lesion. Image clarity score: 3.7. Figure 1B shows the same patient’s vaginal coil T2WI sequence, where the arrow indicates a lesion that is clearer than on the abdominal coil with enhanced contrast. Image clarity score: 4.8.

**Figure 2:**
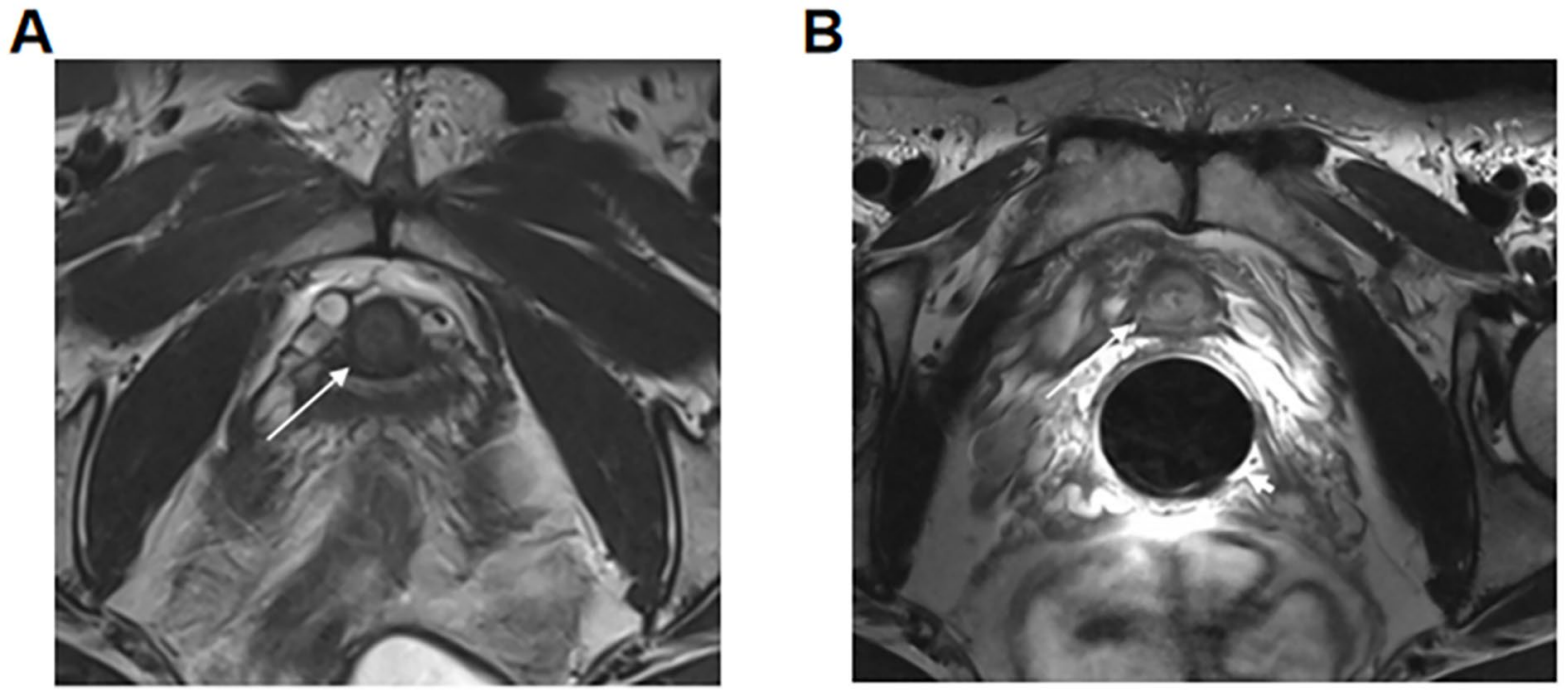
T2-weighted imaging (T2WI) of the same patient. **(A)** Abdominal coil image. The arrow indicates the urethra. **(B)** Vaginal coil image. The long arrow indicates the urethra, and the short arrow points to the coil placed within the vagina.

Due to its vaginal placement, the vaginal coil achieves closer proximity to pelvic organs, enhancing signal acquisition from adjacent structures. This not only provides clear visualization of the cervix but also improves visualization of neighboring organs such as the urethra and rectum, as shown in Figure 2.

**Figure 3:**
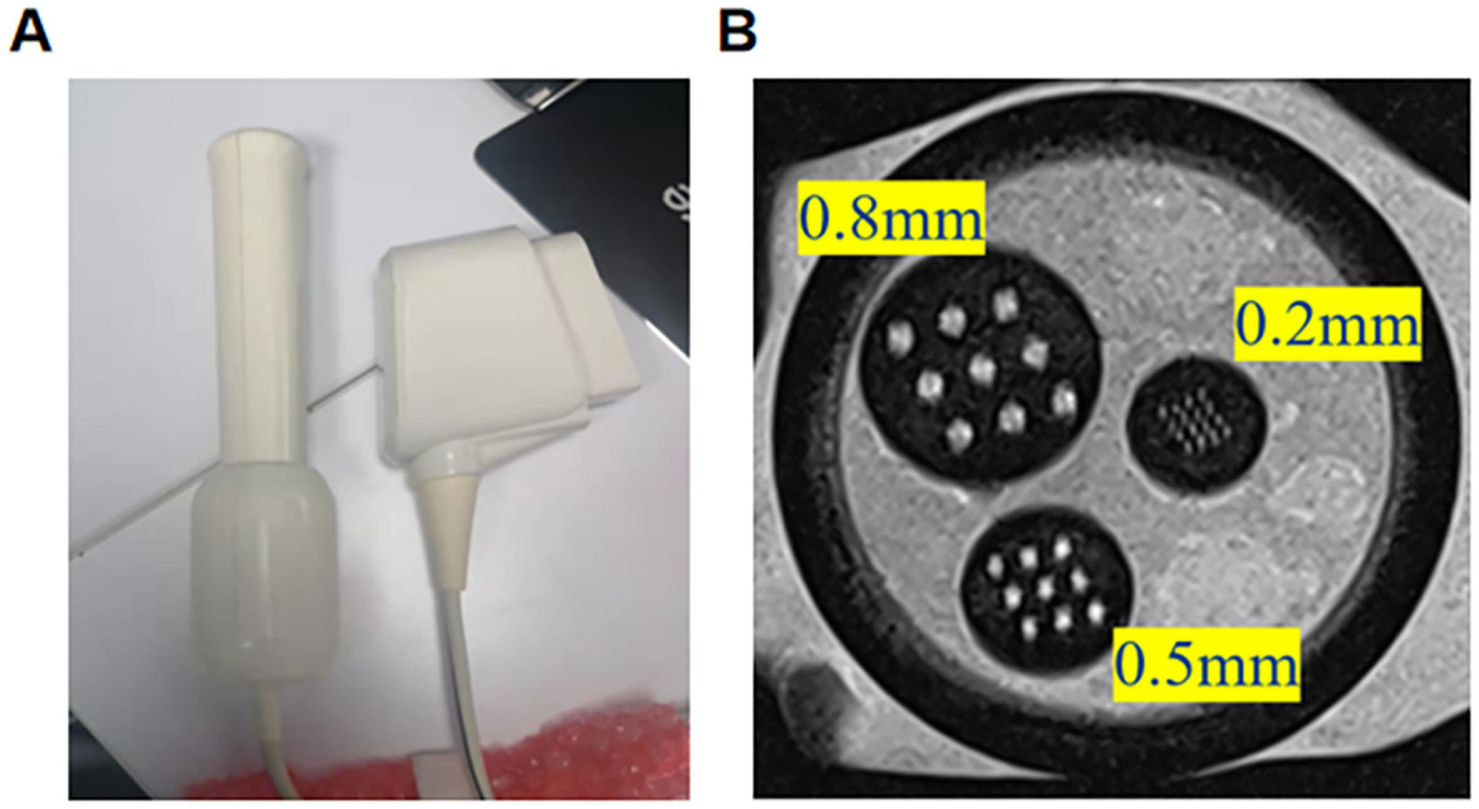
**(A)** Appearance of the vaginal cavity coil. The vaginal insertion portion is on the right, and the connector is on the left. **(B)** In vitro standard model imaging at 3.0T MRI using the vaginal cavity coil. A 0.2 mm diameter hole in the model is clearly visualized.

Figure 2A shows the abdominal coil image with the urethra indicated by an arrow. Figure 2B displays the vaginal coil image from the same patient, where the long arrow indicates the urethra and the short arrow points to the coil placed within the vagina. The vaginal coil image reveals a significantly clearer urethral visualization compared to the abdominal coil, with signal intensity increased by approximately 309.4% (393.80/96.36).Figure 3A shows the appearance of the vaginal cavity coil, with the vaginal insertion portion on the right and the connector on the left. Figure 3B presents *in vitro* standard model imaging at 3.0 T MRI using the vaginal cavity coil, where a 0.2 mm diameter hole in the model is clearly visualized.

## Discussion

Accurate early staging of cervical cancer is crucial for guiding clinical treatment decisions, particularly for selecting fertility-preserving surgical options in patients with childbearing desires (3). This study evaluated the diagnostic value of 3.0 T MRI combined with a vaginal coil in preoperative staging of early cervical cancer (FIGO stages Ia1-Ib1). Results demonstrated that compared to the conventionally used body phased-array coil, the vaginal coil significantly improved cervical local image quality and enhanced the accuracy of preoperative staging.

FIGO staging of cervical cancer heavily relies on precise assessment of tumor size and local invasion depth. While conventional body coil MRI provides excellent panoramic views of pelvic anatomy, its inherent spatial resolution and signal-to-noise ratio limitations hinder detection of early microinvasion into the cervical stroma (5). This study demonstrates that placing a vaginal coil adjacent to the cervix significantly enhances the visualization clarity of critical anatomical structures including the cervical mucosa, stroma, and muscle layers. This finding aligns with Zhang et al.’s (6) quantitative analysis confirming that intravaginal coil imaging markedly improves image signal-to-noise ratio and contrast-to-noise ratio, thereby providing technical assurance for visualizing cervical microstructures. This improvement in imaging quality directly translates to enhanced clinical diagnostic performance: in this study, the vaginal coil significantly increased the overall accuracy of preoperative MRI staging for early cervical cancer from 37.5% with a body coil to 82.5%, demonstrating particularly higher sensitivity in identifying smaller Ia1 stage lesions. This finding further supports the earlier conclusion by deSouza et al. (7) that intraluminal coil technology offers higher detection sensitivity for small-volume cervical cancers.

Concurrently, this study objectively highlights the limitations of the intravaginal coil technique. Its imaging advantages are primarily confined to the cervical region immediately adjacent to the coil, whereas visualization of relatively distant pelvic structures (such as the posterior bladder wall and anterior rectal wall) is inferior to that achieved by the broader coverage of body coils. This reflects the physical characteristics of near-field coil technology. Therefore, in clinical practice, the endocervical coil should be considered a valuable supplement to conventional body coil scanning rather than a replacement. It is suitable for targeted imaging of localized cervical lesions with unclear borders on conventional MRI images or those requiring precise assessment of invasion depth (4). This study employed an optimized high-resolution scanning sequence, particularly combining thin-slice T2WI with ZOOMit-DWI technology. The latter effectively reduces gas and fat-induced susceptibility artifacts in the pelvis, thereby enhancing the image quality and diagnostic value of diffusion-weighted imaging in the cervical region, consistent with findings by Downey et al. (8).

The findings of this study hold clear clinical translational significance. For young patients with early-stage cervical cancer who desire fertility preservation, accurately assessing whether the tumor has invaded the deep cervical stroma is the core basis for determining the feasibility of fertility-sparing procedures such as cervical conization or radical cervical resection (9). The high spatial resolution images provided by the vaginal coil offer clinicians more reliable radiographic evidence regarding tumor depth of invasion. This facilitates the development of more individualized and precise surgical plans, maximizing fertility preservation while ensuring oncological safety.

This study has several limitations. First, the sample size was relatively small, and as a single-center retrospective study, the generalizability of its conclusions requires validation through larger prospective studies. Second, the study focused exclusively on early-stage (Ia1-Ib1) cases, failing to evaluate the diagnostic efficacy of this technique for more advanced cervical cancers (e.g., assessing parametrial invasion or lymph node metastasis). Additionally, intra-cavity coil placement requires patient cooperation and has specific contraindications. Future research should include multicenter collaborations to expand sample size and explore integrating high-resolution imaging features from intra-cavity coils with radiomics or artificial intelligence analysis models to develop more robust preoperative prediction tools. Fourth, while our post-hoc power analysis indicated adequate power (0.86) to detect the observed 45% difference in staging accuracy between coil groups, the modest sample size (*n* = 40) resulted in relatively wide confidence intervals for some subgroup analyses (e.g., stage-specific accuracy). Larger prospective studies are warranted to provide more precise estimates of diagnostic performance, particularly for rare histological subtypes and specific FIGO substages.

## Conclusion

In summary, 3.0 T MRI combined with vaginal intrauterine coil placement significantly enhances local image quality for early-stage cervical cancer and substantially improves the accuracy of preoperative FIGO staging. This technique serves as a valuable adjunct to conventional body coil MRI examinations, providing critical imaging support for personalized precision treatment of early cervical cancer—particularly for fertility-sparing surgical decisions—and demonstrates promising clinical application prospects.

## Data Availability

The original contributions presented in the study are included in the article/supplementary material, further inquiries can be directed to the corresponding author.
